# P74. A Good Manufacturing Practice procedure to generate therapeutic numbers of highly pure anti-leukaemic virus-specific T-cells

**DOI:** 10.1186/2051-1426-2-S2-P48

**Published:** 2014-03-12

**Authors:** MM van Loenen, R de Boer, E van Liempt, RS Hagedoorn, P Meij, I Jedema, JHF Falkenburg, MHM Heemskerk

**Affiliations:** 1 Leiden University Medical Center, Hematology, Leiden, the Netherlands; 2 Leiden University Medical Center, Clinical Pharmacy and Toxicology, Leiden, the Netherlands

## Background

Recently, we have started a clinical trial to treat patients with high risk acute leukaemia with a donor-derived HA-1-TCR transduced virus-specific T-cell product as early as 8 weeks and 14 weeks after allogeneic stem cell transplantation (allo-SCT). Donor derived Cytomegalovirus (CMV)- and Epstein Bar virus (EBV)-specific T-cells will be isolated using Streptamer based CliniMACS selection, and will be subsequently transduced at day 2 with the well-characterized anti-leukaemic HA-1-TCR and infused 10-12 days later. Based on these well-defined specificities this T-cell product is predicted to result in a selective Graft versus Leukaemia (GvL) effect without Graft versus Host Disease (GvHD). Important study parameters are persistence of the T-cell product, feasibility of generation of HA-1-TCR transduced virus-specific T-cells, and the number of events of acute GvHD.

## Material and methods

To obtain therapeutic cell numbers, one of the inclusion criteria is presence in donor peripheral blood of 1 or 2 virus-specific T-cell population with a frequency of ≥0.05% of T-cells. MHC-Streptamers will be used to isolate 1 or 2 virus-specific T-cell populations from donor leukocytes. MHC-Streptamer incubation will result in binding of the TCR of the virus-specific T-cells of interest to the specific peptide presented by the MHC molecule on the Streptamers. Next to allowing selection of T-cells of interest, this binding will also result in specific stimulation allowing subsequent transduction with the HA-1-TCR. The process of isolation of pure populations of virus-specific T-cells and transduction with good manufacturing practice (GMP)-grade retroviral supernatant encoding the HA-1-TCR has been validated with 4 large scale test procedures in the cleanroom. To pass the in process (IP) testing, T-cells needed to be ≥50% pure for the respective virus-specific tetramer directly after Streptamer isolation. In addition, after transduction and subsequent culturing T-cells need to be ≥60% antigen-specific, as measured with virus- and HA-1-tetramers. Moreover, transduction efficiency of ≥5% as measured with HA-1-tetramers is a prerequisite.

## Results

All HA-1-TCR td virus-specific T-cell products met the criteria for IP testing and quality control testing. They contained >90% antigen-specific T-cells and >10% HA-1 tetramer positive T-cells. Moreover, all HA-1-TCR td virus-specific T-cell products were highly reactive against HA-1-positive leukaemic cells.

## Conclusions

Here, we present a GMP-grade procedure to generate in a short culture period of less than 2 weeks therapeutically relevant numbers of defined antigen-specific and highly anti-leukaemia reactive T-cells.

